# Supplementary material for: Insightful Improvement in the Design of Potent Uropathogenic E. coli FimH Antagonists
Source: Pharmaceutics. 2023 Feb 4;15(2):527. doi: 10.3390/pharmaceutics15020527 (PMC9962304; doi:10.3390/pharmaceutics15020527)
Supplement: Supplementary file 1 [file pharmaceutics-15-00527-s001.zip › pharmaceutics-2182684-supplementary.pdf]

FURTHER INSIGTHS IN THE DESIGN OF POTENT UROPATHOGENIC *E. COLI* FIMH ANTAGONISTS

Leila Mousavifar[1], Meysam Sarshar[2], Clarisse Bridot[3], Daniela Scribano[4], Cecilia Ambrosi[5,6], Anna Teresa Palamara[4,7], Gerard Vergoten[8], Benoît Roubinet[9], Ludovic Landemarre[9], Julie Bouckaert[3]*, René Roy[1]*

[1] Glycosciences and Nanomaterial Laboratory, Université du Québec à Montréal, P.O. Box 8888, Succ. Centre-Ville, Montréal, Québec H3C 3P8, Canada
[2] Research Laboratories, Bambino Gesù Children's Hospital, IRCCS, 00146 Rome, Italy
[3] Unité de Glycobiologie Structurale et Fonctionnelle (UGSF), UMR8576 du CNRS, Univ. Lille, F- 59000, Lille, France
[4] Department of Public Health and Infectious Diseases, Sapienza University of Rome, 00185 Rome, Italy
[5] Department of Human Sciences and Promotion of the Quality of Life, San Raffaele Roma Open University, 00166 Rome, Italy
[6] IRCCS San Raffaele Roma, Rome, Italy
[7] Department of Infectious Diseases, National Institute of Health, 00161 Rome, Italy
[8] University of Lille, Institut de Chimie Pharmaceutique Albert Lespagnol (ICPAL), Faculté de Pharmacie, 3 rue du Professeur Laguesse, BP-83, F-59006, Lille, France
[9] GLYcoDiag, 2 rue du Cristal, 45100 Orléans, France
* Correspondence: roy.rene@uqam.ca ; julie.bouckaert@univ-lille.fr

Materials and Methods

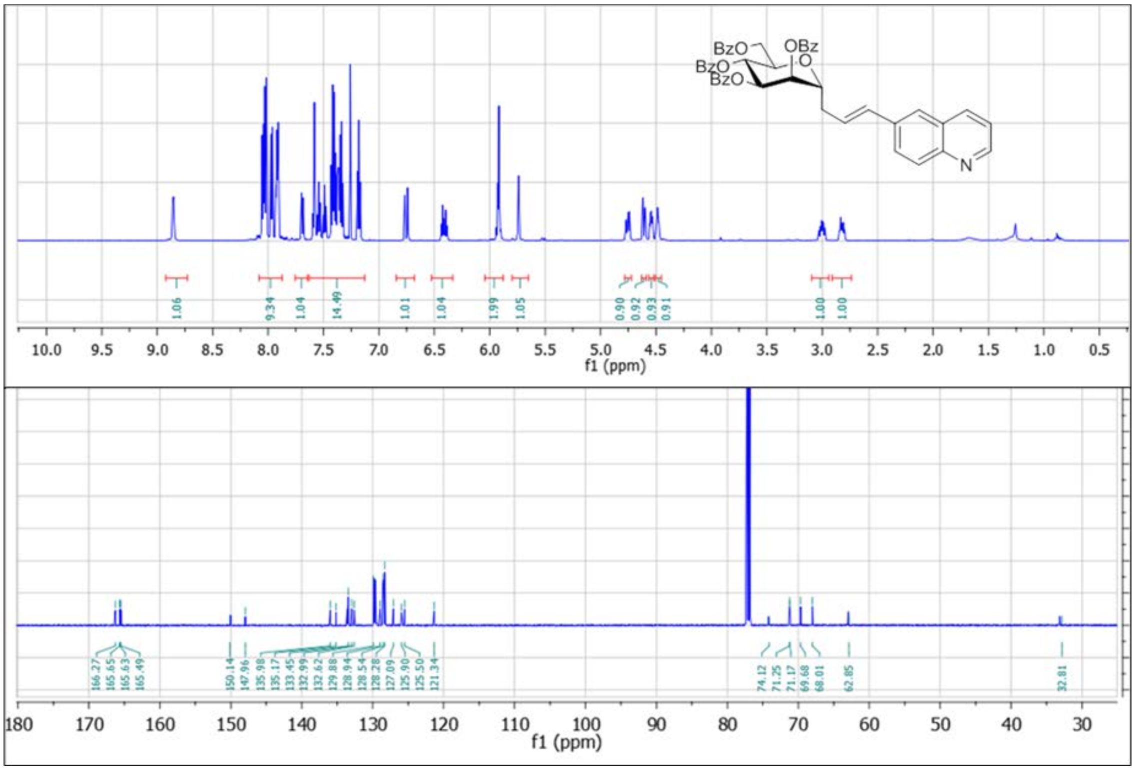

Figure S1. ^{1}HNMR and ^{13}CNMR of compound 2 ($CDCl_3$, 600 and 151 MHz, respectively)

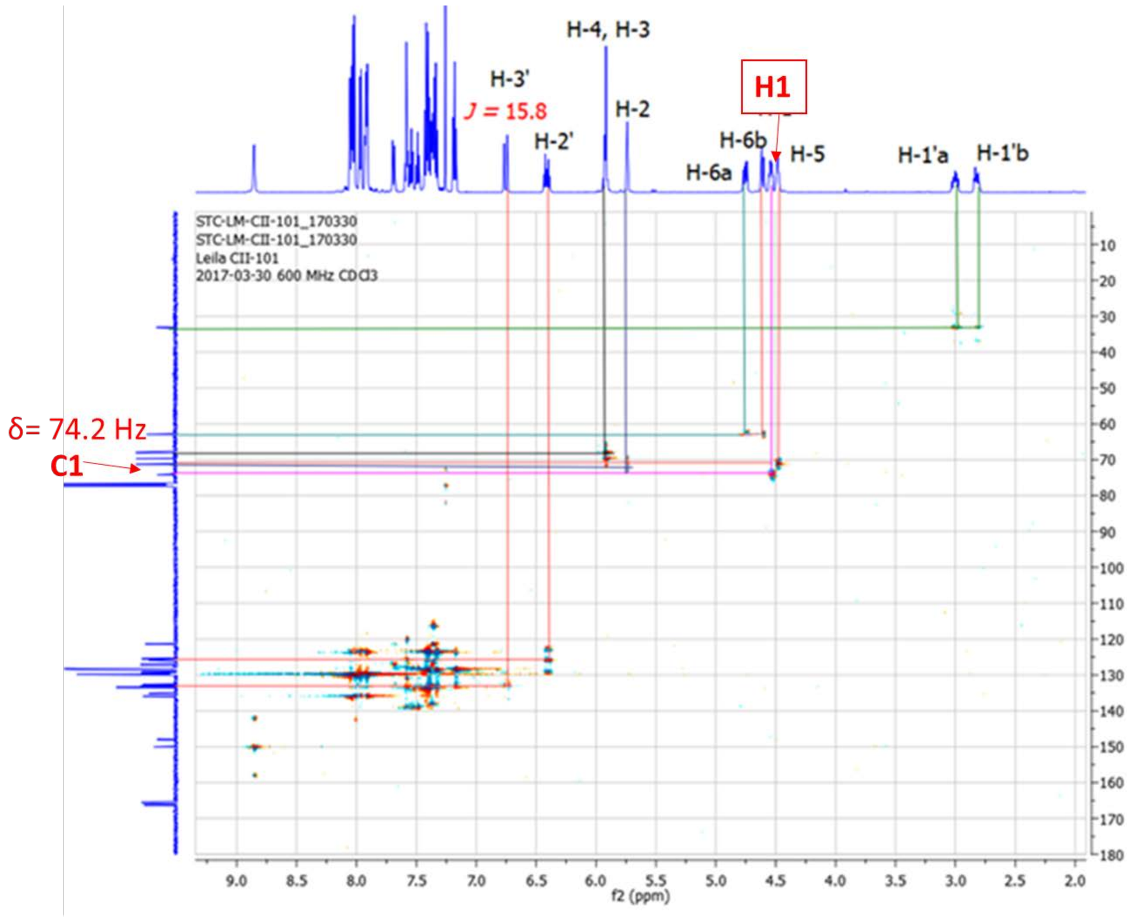

Figure S2. HSQC of purified compound 2. The coupling constant of H-3′ (J2′,3′=15.8 Hz) indicates the presence of the trans stereoisomer. (CDCl3, 600 MHz)

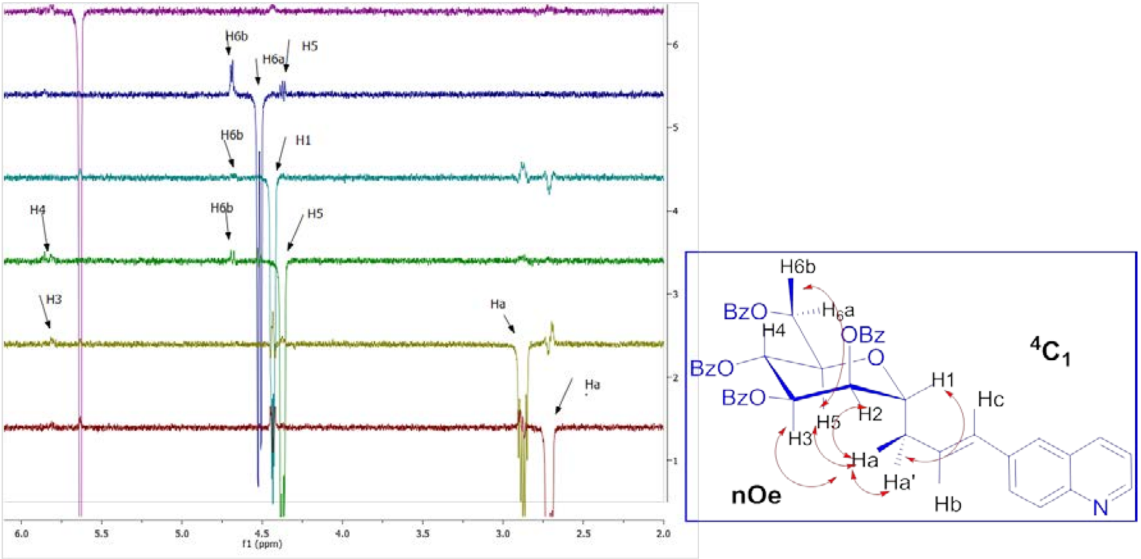

Figure S3. Conformational studies of compound 2 in solution using NOESYtechnique 600 MHz.

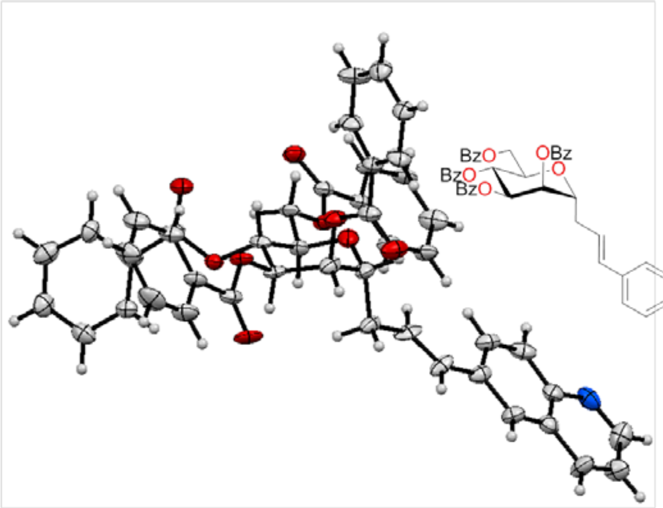

Figure S4. The ORTEP diagram of compound 2 clearly shows it to exist as the proper chair conformer *4C1.* X-Ray crystal-structure and refinement data: formula, ($C_{46}H_{37}NO_9$), monoclinic, space group P21, a =24.6459 (12) Å, b 6.3450 (3) Å, c 25.5657 (12) Å, β= 108.871(2)°, 90, V =3783.0(3) Å3,Z=4,T=150K, Dcalcd 1.313 g/cm3. Crystallographic data for the structure reported in this paper has been deposited at the Cambridge Crystallographic Data Centre (CCDC) with deposition no: (CCDC:1560371). Supplementary data can be obtained free of charge from CCDC, 12 Union Road, Cambridge CB2 1EZ,

UK (fax: (+44)1223-336-033; e-mail: deposit@ccdc.cam.ac.uk

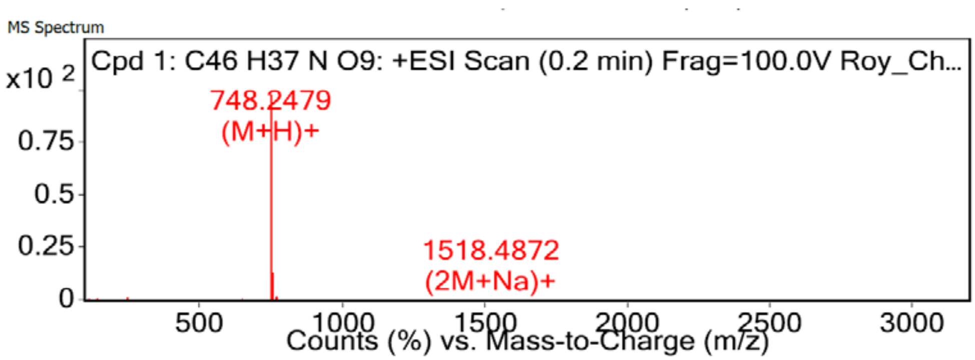

Figure S5. ESI+-HRMS spectrum of compound 2

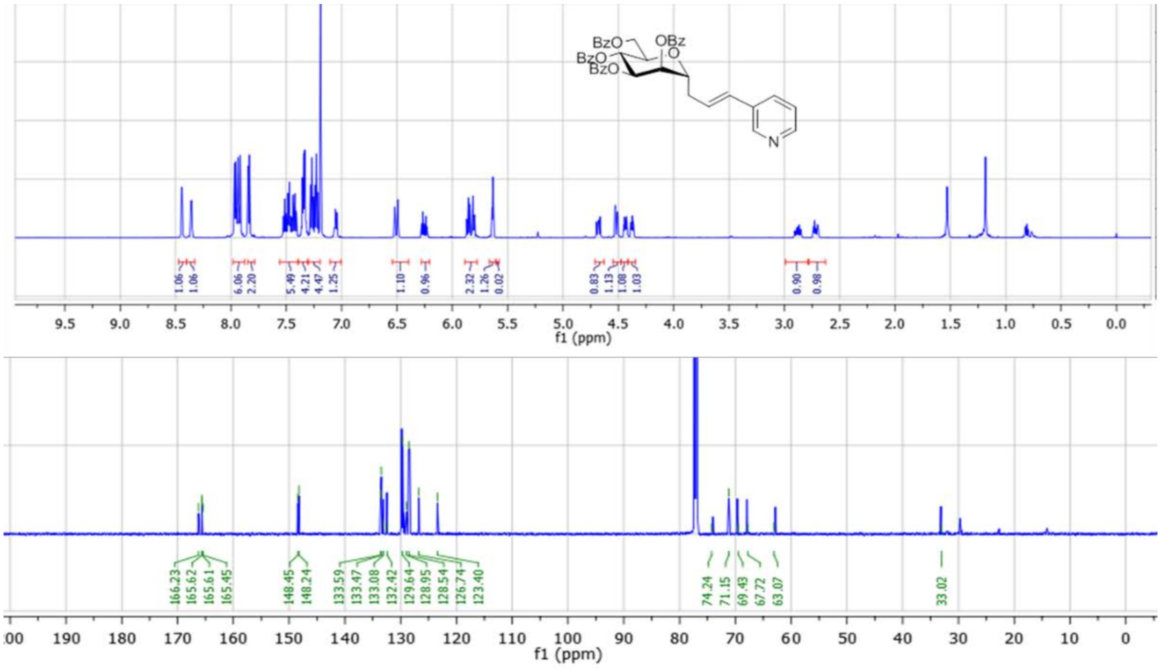

Figure S6. ^{1}HNMR and ^{13}CNMR of compound 3(CDCl3, 600 a nd 151 MHz, respectively)

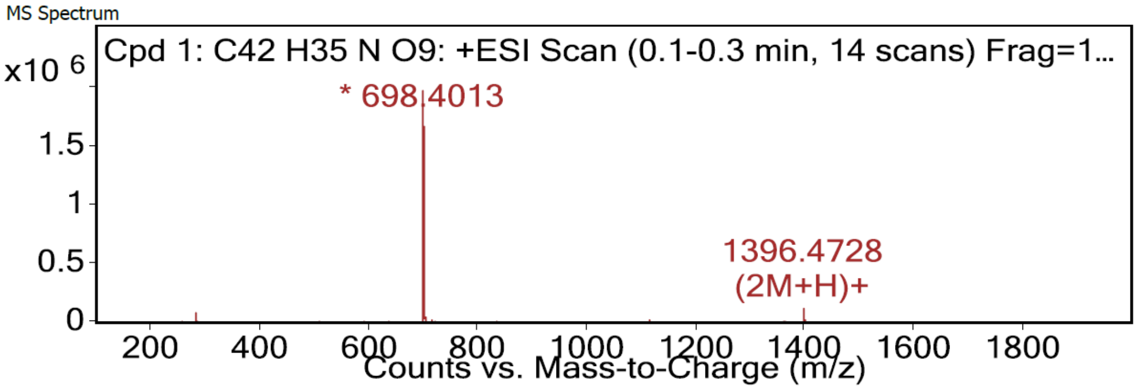

Figure S7. ESI+-HRMS spectrum of compound 3

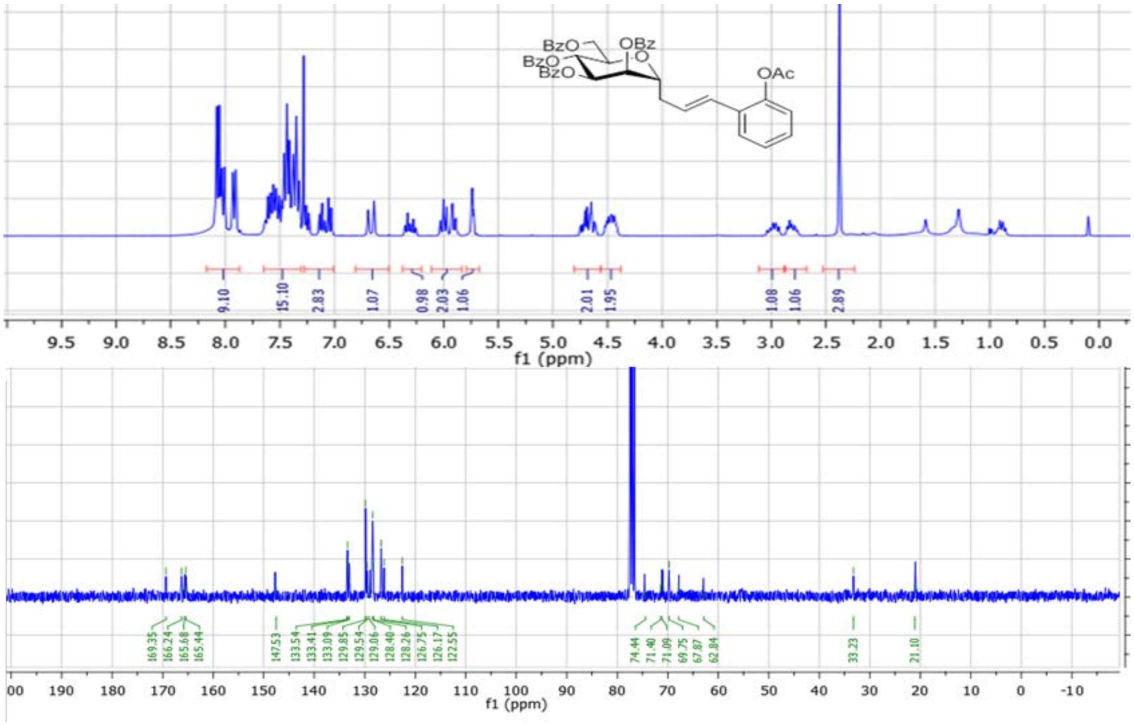

Figure S8. ^{1}HNMR and ^{13}CNMR of compound 4($CDCl_3$, 300 and 75 MHz respectively)

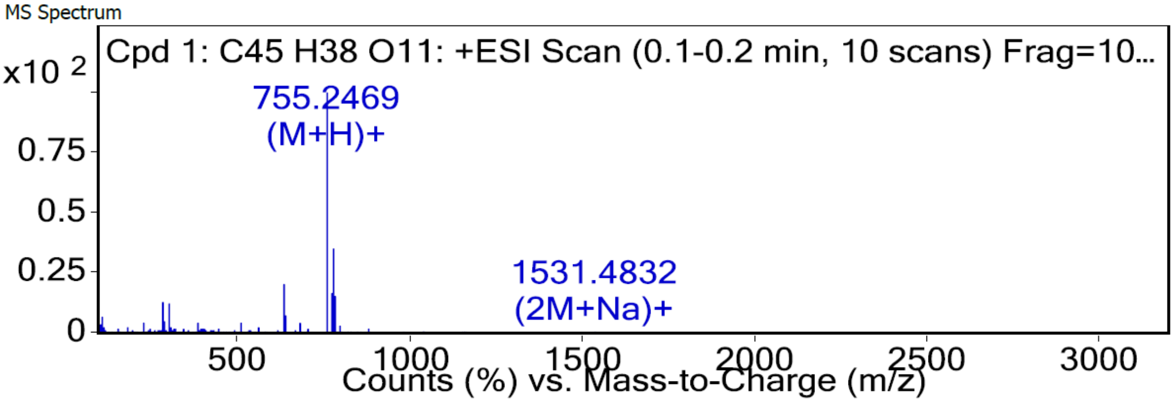

Figure S9. ESI+-HRMS spectrum of compound 4

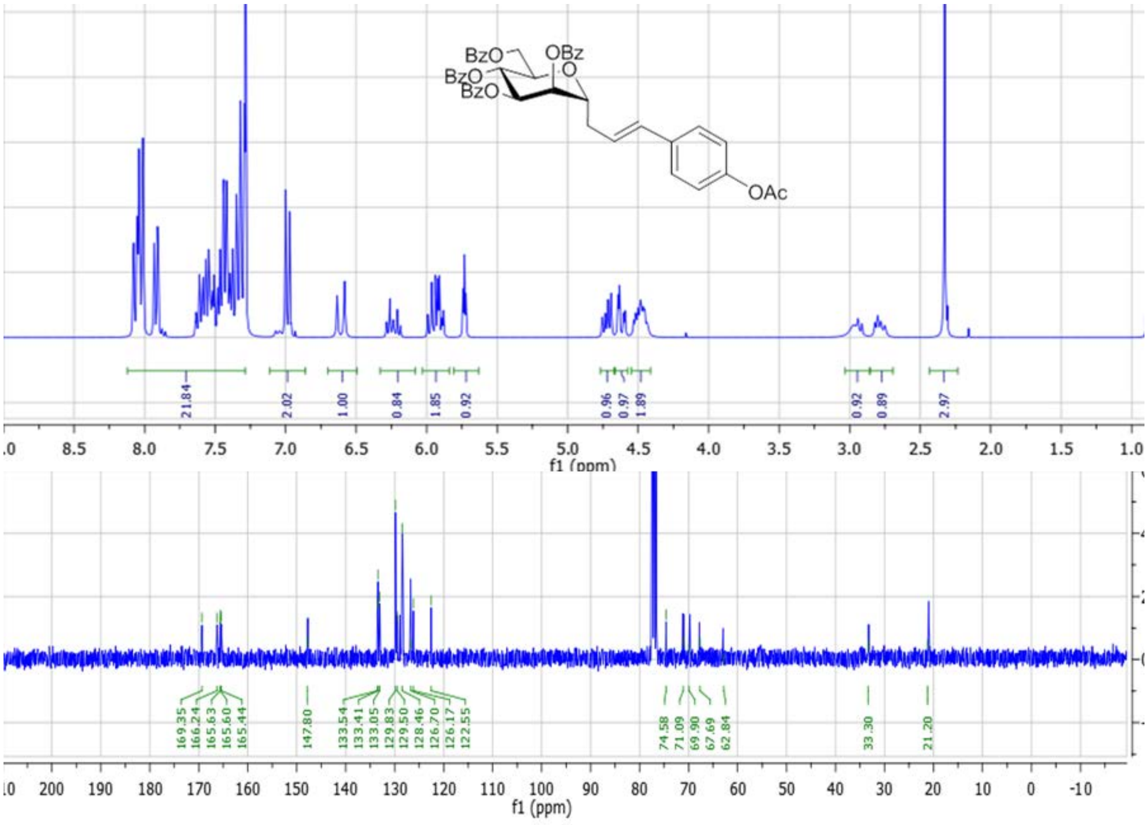

Figure S10. ^{1}HNMR and ^{13}CNMR of compound 5(CDCl3, 300 and 75 MHz respectively)

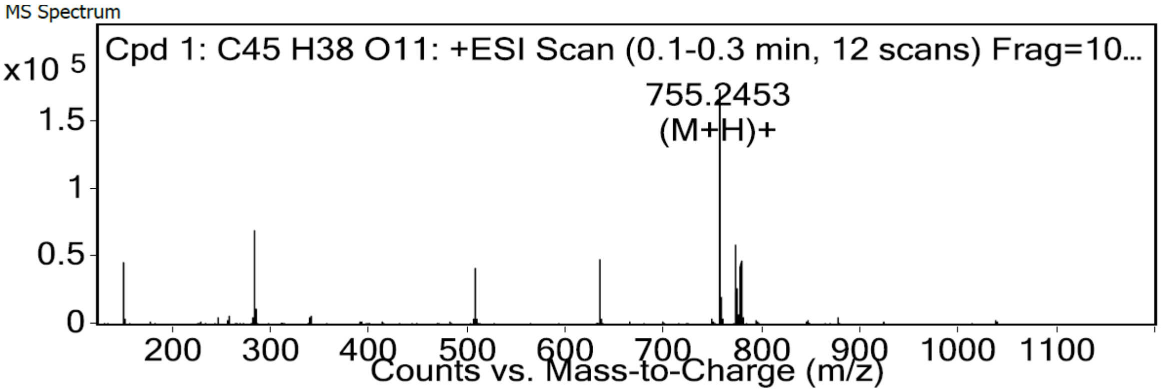

Figure S11. ESI+-HRMS spectrum of compound 5

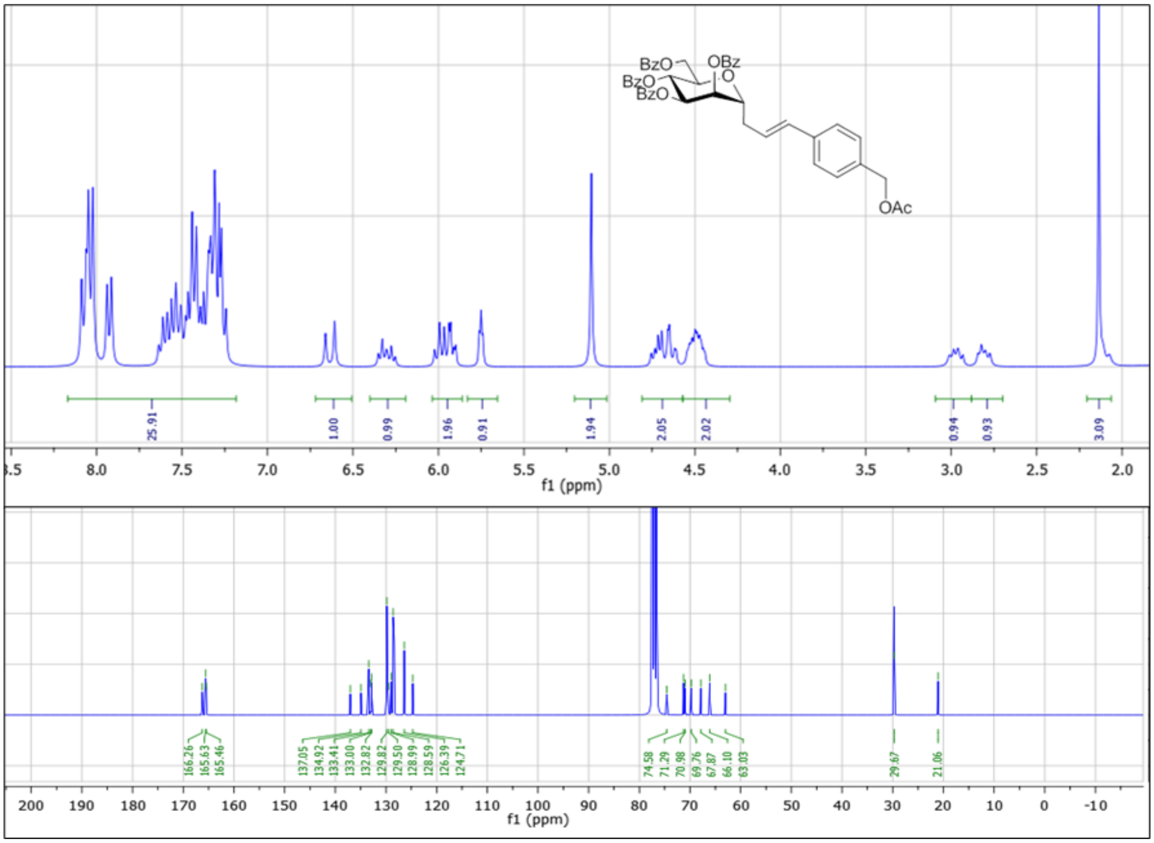

Figure S12. ^{1}HNMR and ^{13}CNMR of compound 6(CDCl$_3$, 300 and 75 MHz respectively)

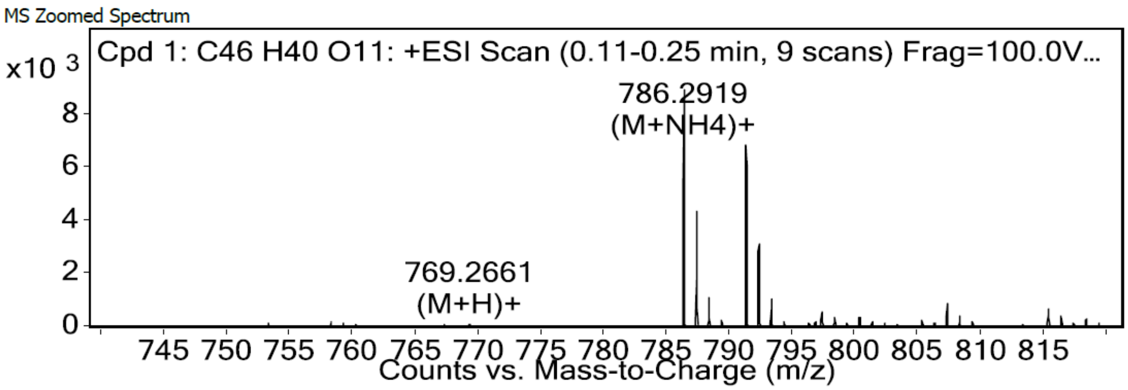

Figure S13. ESI+-HRMS spectrum of compound 6

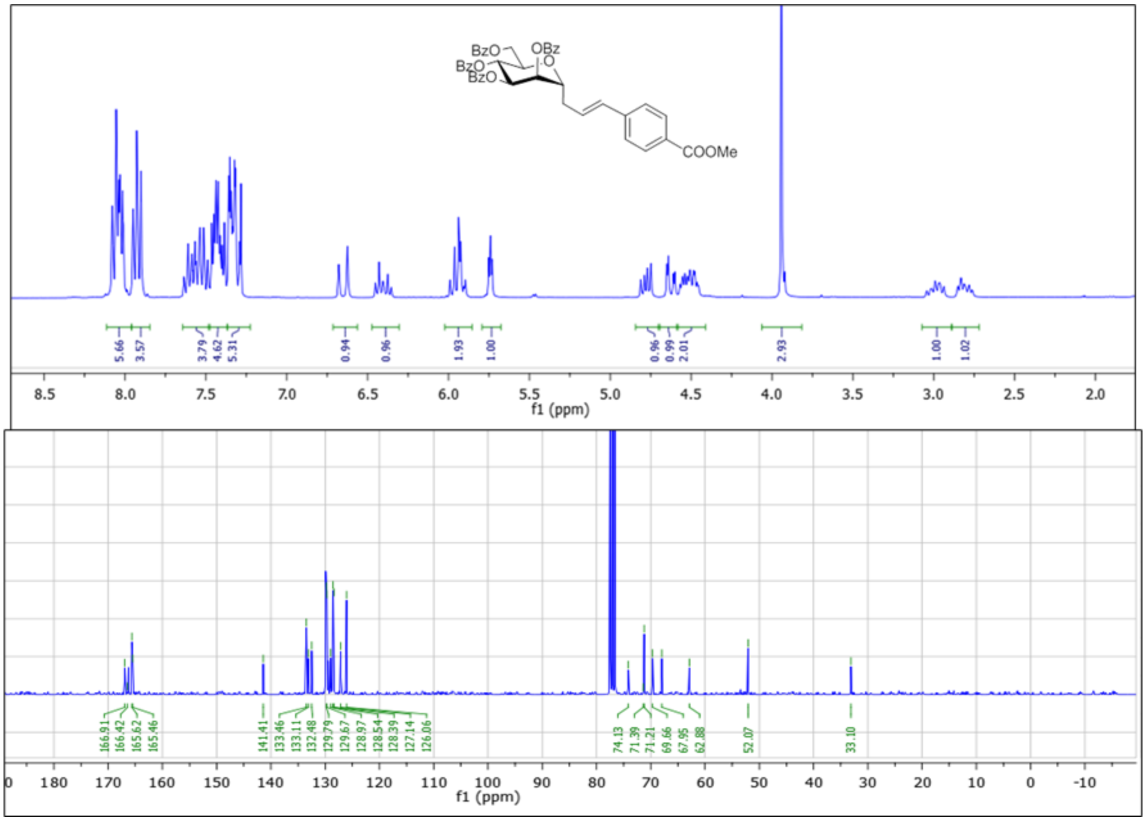

Figure S14. ^{1}HNMR and ^{13}CNMR of compound 7(CDCl3, 300 and 75 MHz respectively)

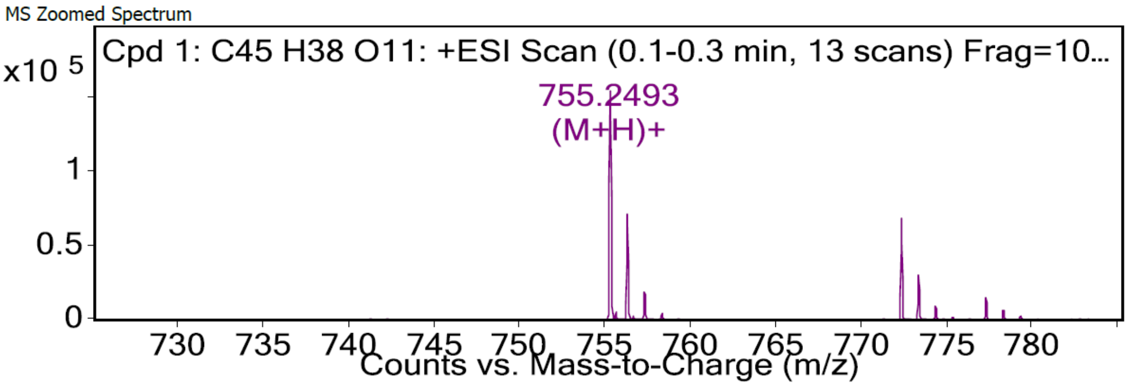

Figure S15. ESI+-HRMS spectrum of compound 7

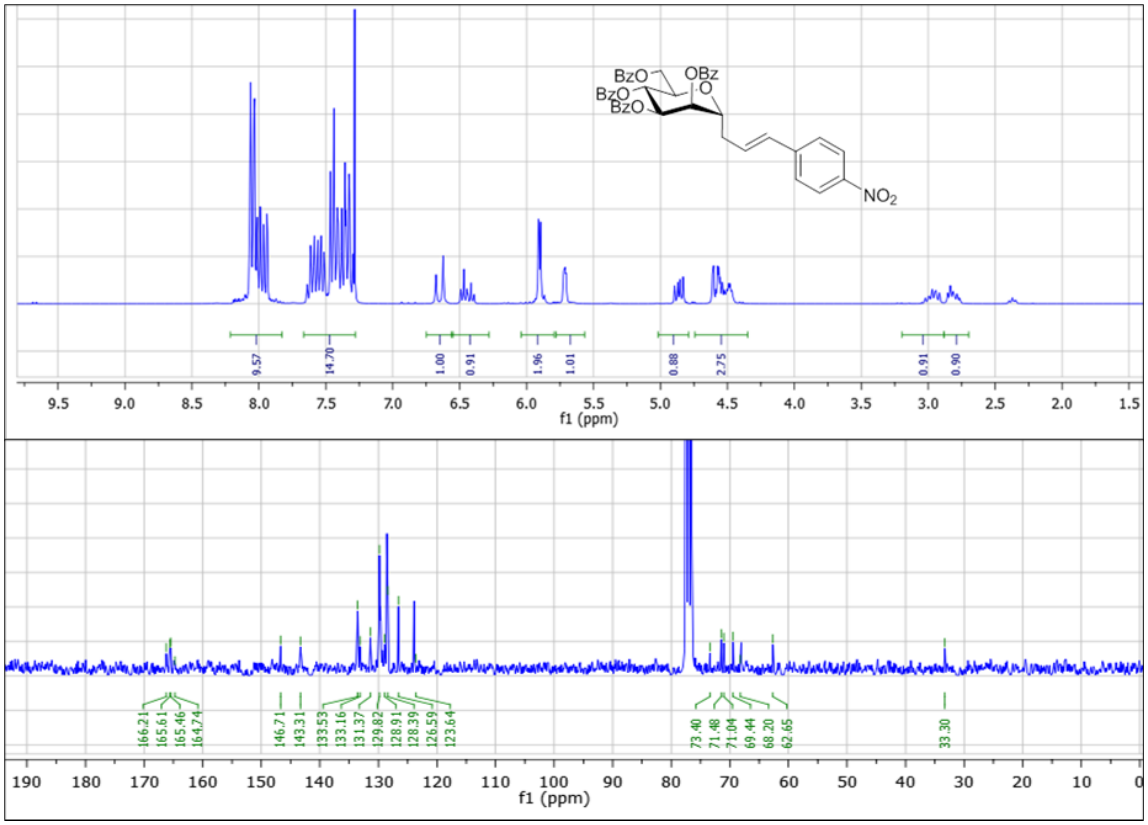

Figure S16. ^{1}HNMR and ^{13}CNMR of compound 8($CDCl_3$, 300 and 75 MHz respectively)

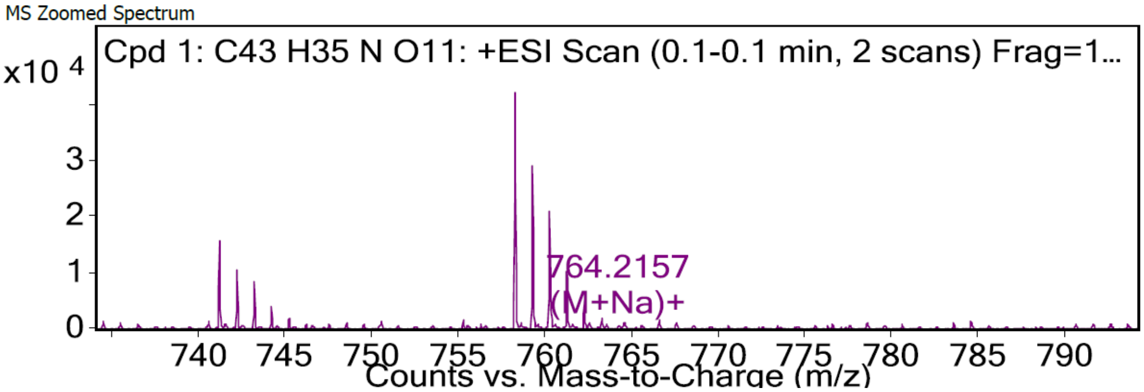

Figure S17. ESI+-HRMS spectrum of compound 8

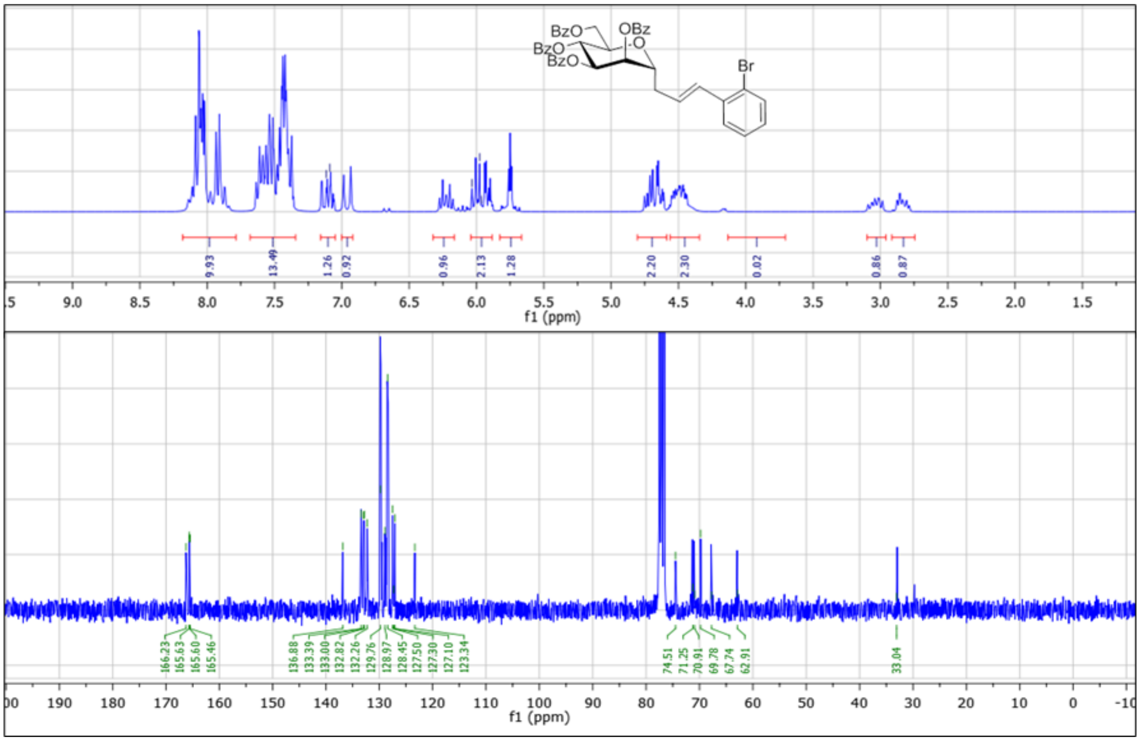

Figure S18. ^{1}HNMR and ^{13}CNMR of compound 10($CDCl_3$, 300 and 75 MHz respectively)

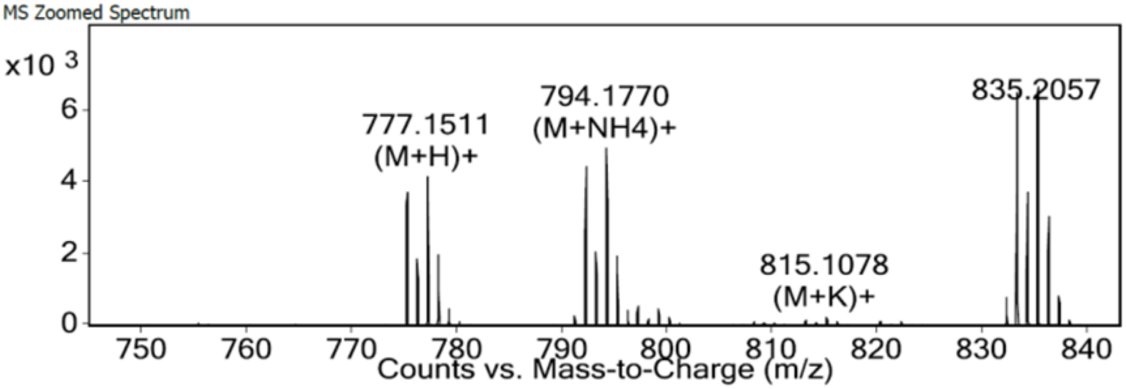

Figure S19. ESI+-HRMS spectrum of compound 10

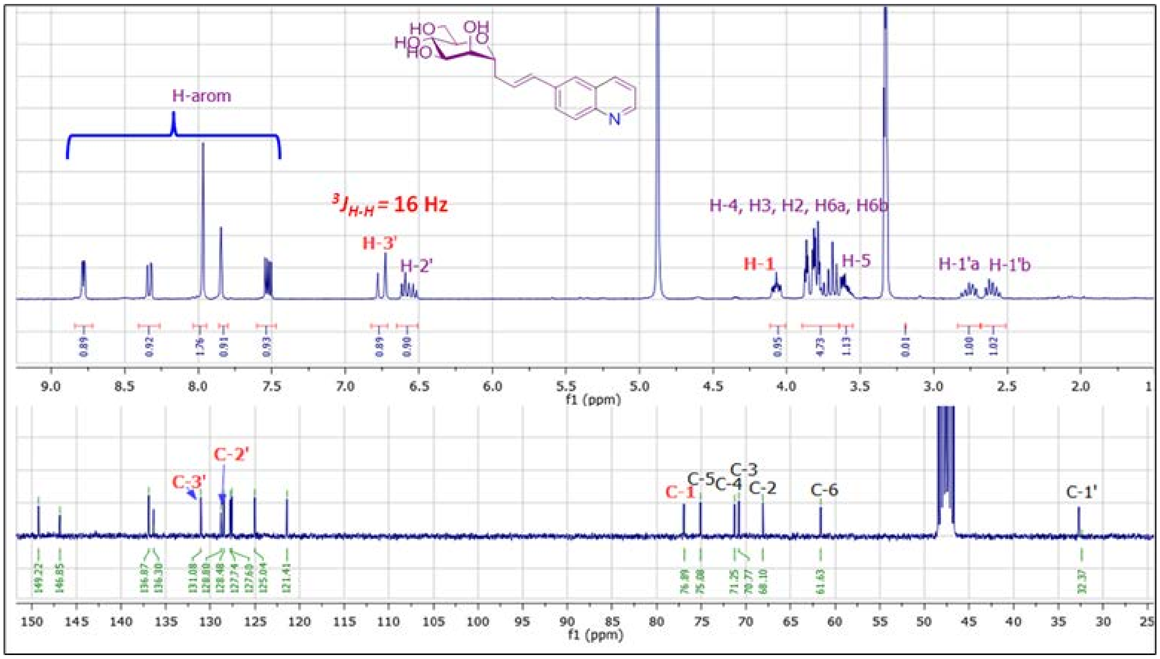

Figure S20. Fully assigned ^{1}HNMR and ^{13}CNMR of compound 11(CD_3OD, 600 and 151MHz respectively)

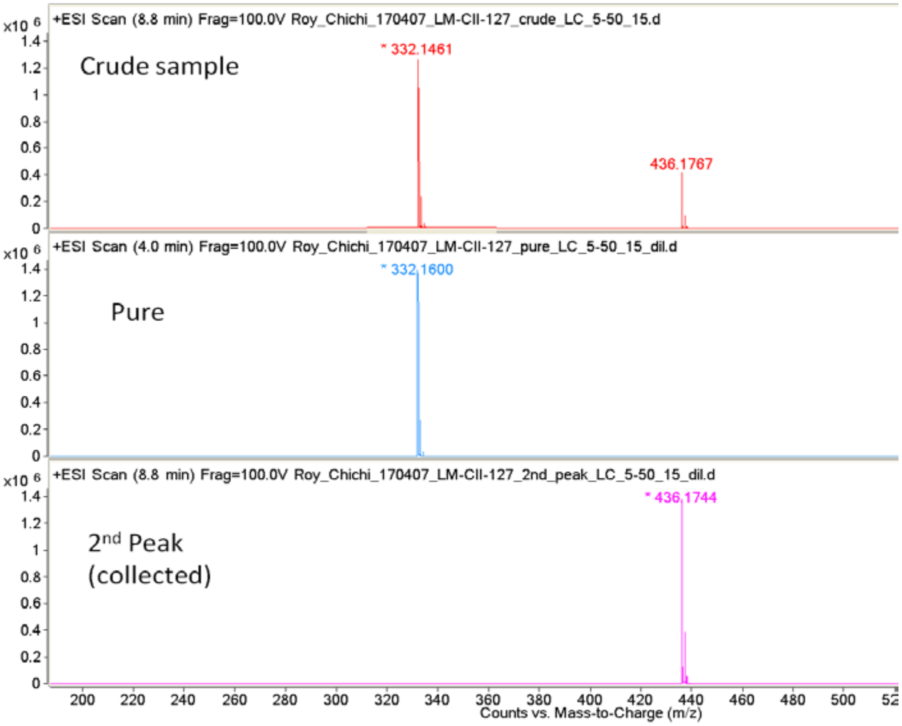

Figure S21. HPLC-TOF-MAS analysis spectrum of compound 11

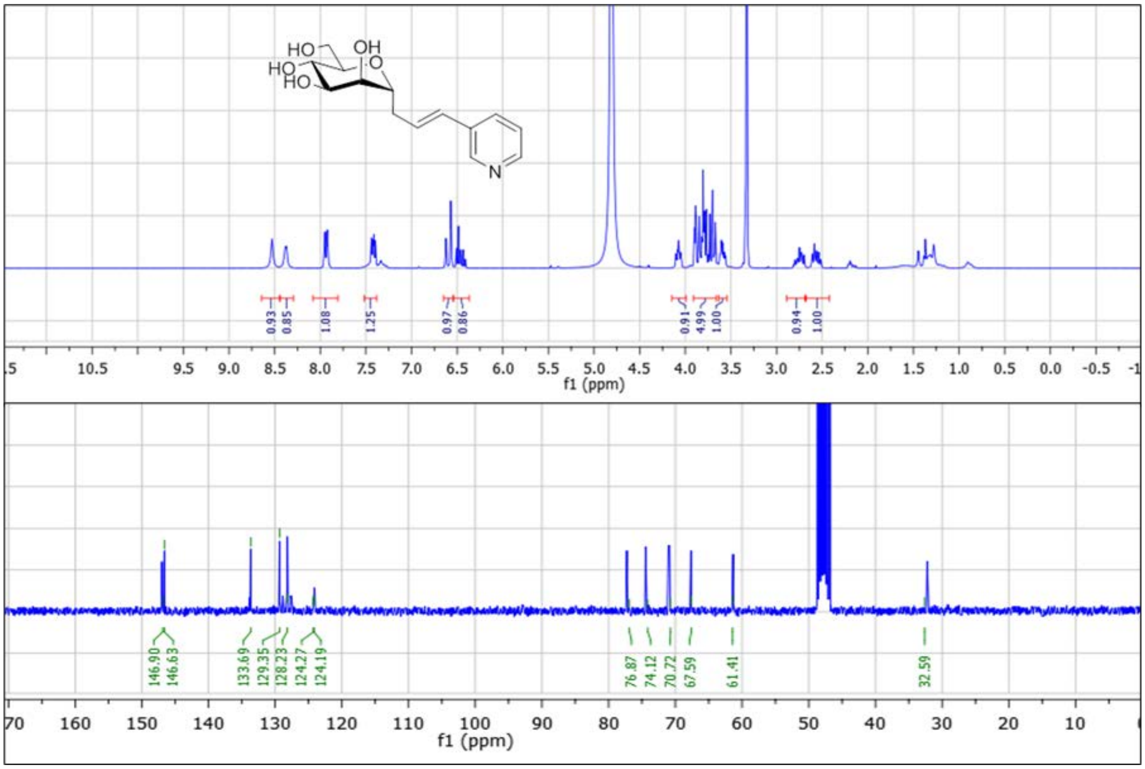

Figure S22. ^{1}H NMR and ^{13}CNMR spectrum of compound 12 (CD_3OD, 300 and 75MHz respectively)

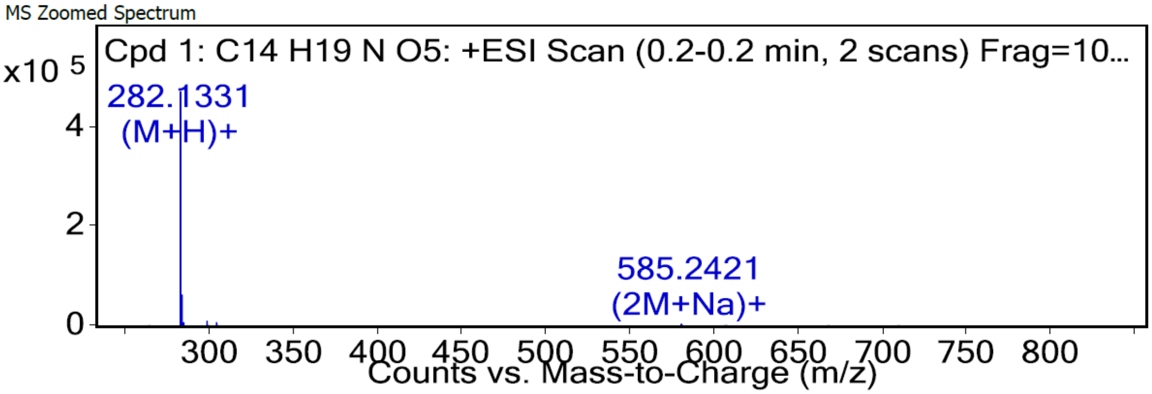

Figure S23. ESI+-HRMS spectrum of compound 12

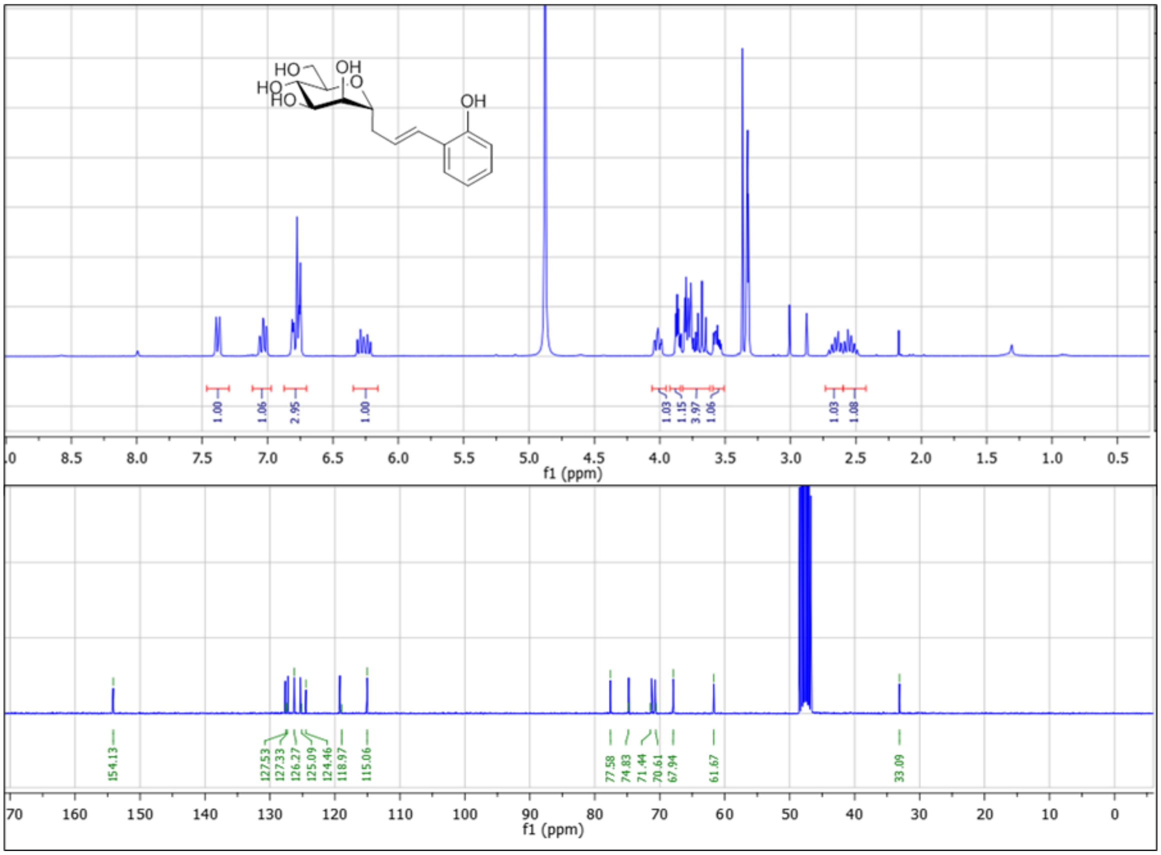

Figure S24. ^{1}H NMR and ^{13}CNMR spectrum of compound 13(CD$_3$OD, 300 and 75MHz respectively

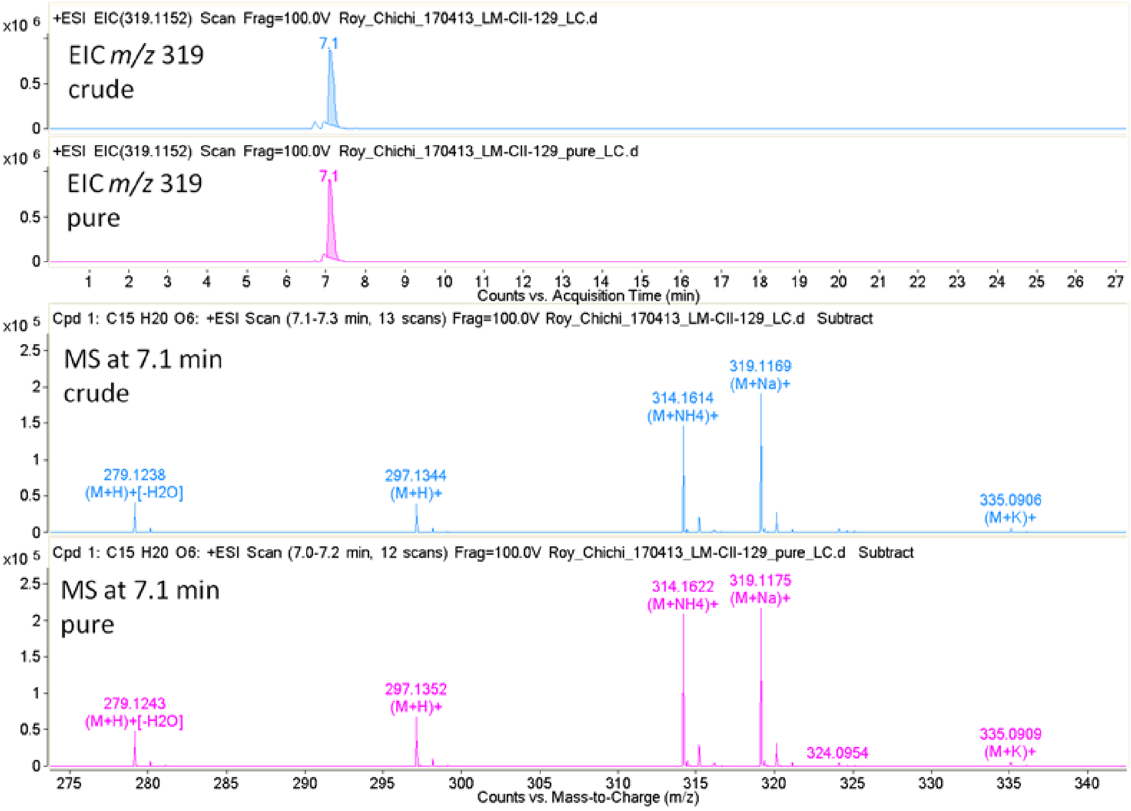

Figure S25. HPLC-TOF-MAS analysis spectrum of compound 13

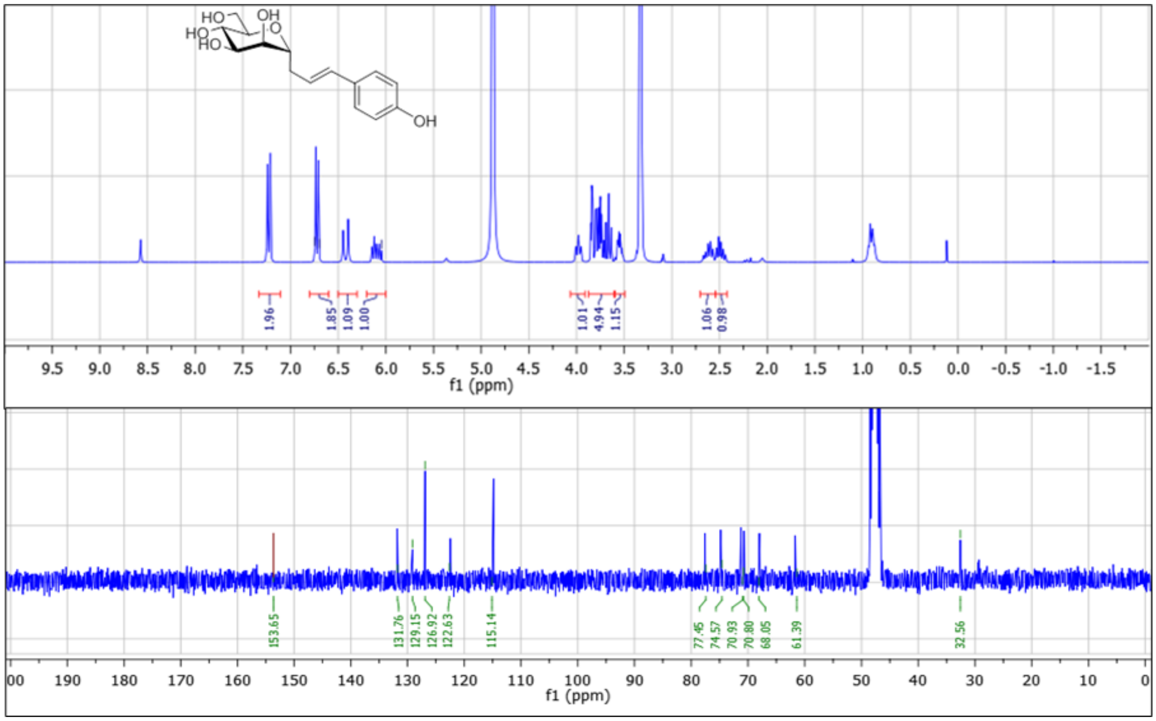

Figure S26. ¹H NMR and ¹³CNMR spectrum of compound 14 (CD_3OD, 300 and 75MHz respectively

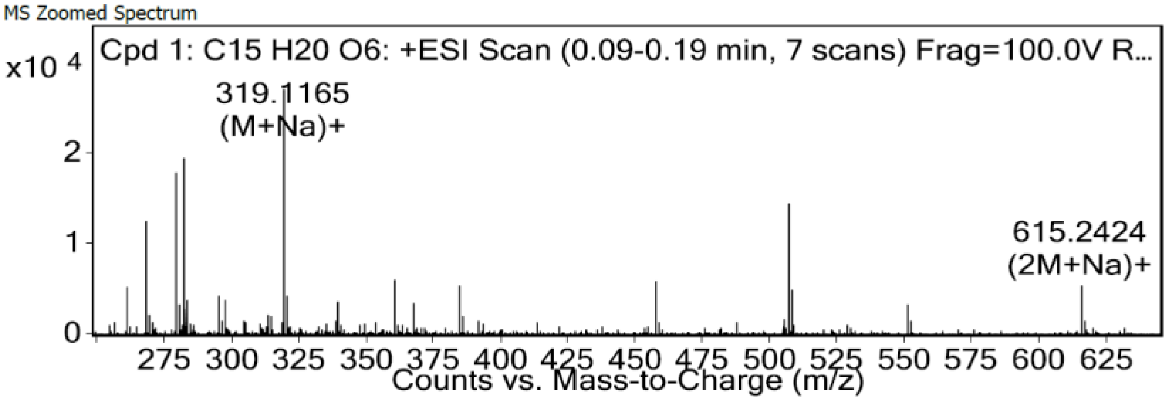

Figure S27. ESI+-HRMS spectrum of compound 14

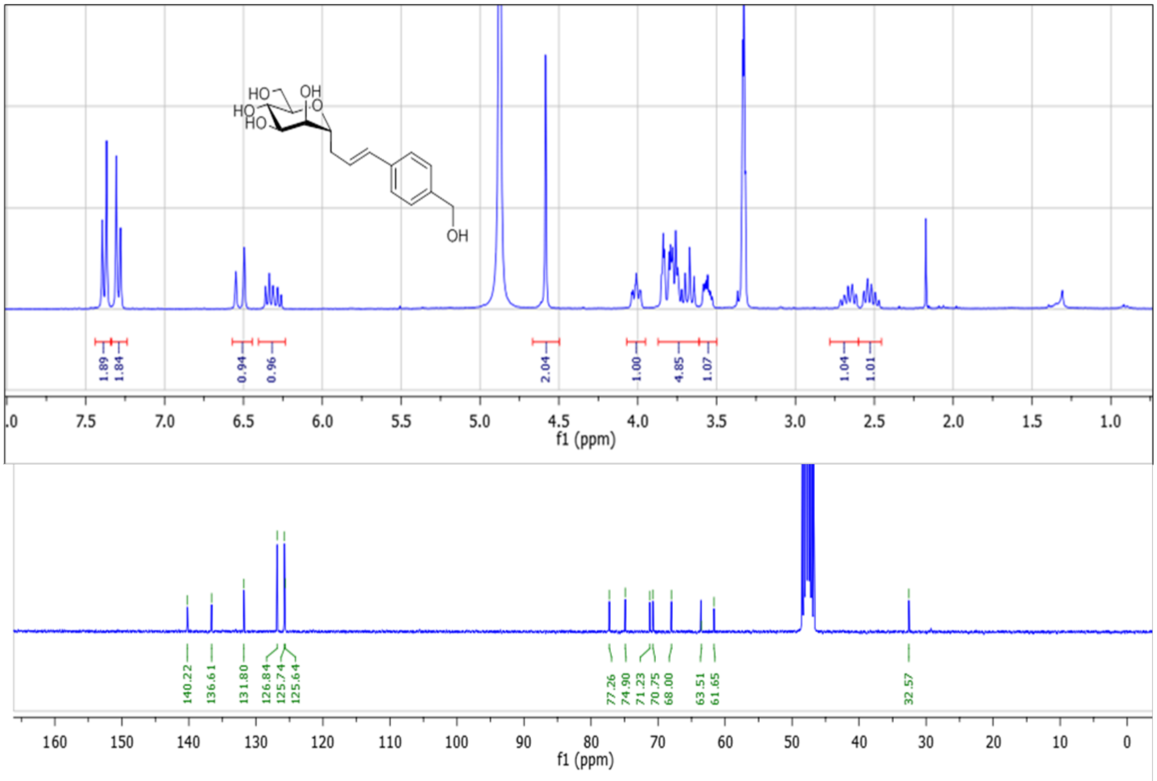

Figure S28. ¹H NMR and ¹³CNMR spectrum of compound 15 (CD_3OD, 300 and 75MHz respectively

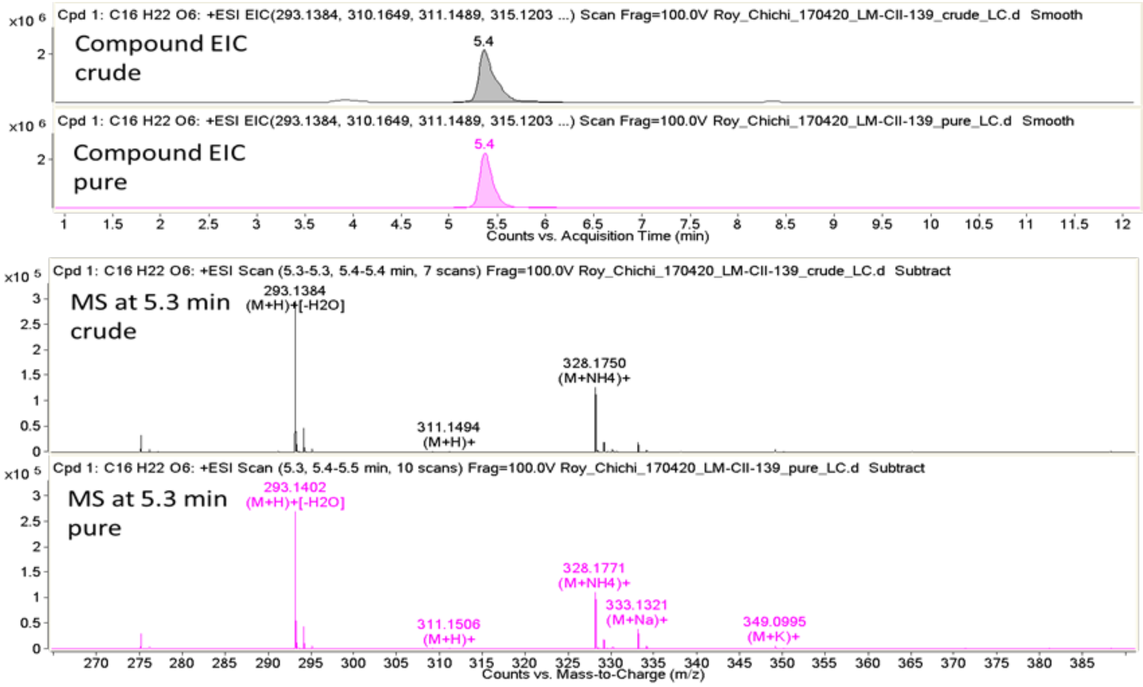

Figure S29. HPLC-TOF-MAS analysis spectrum of compound 15

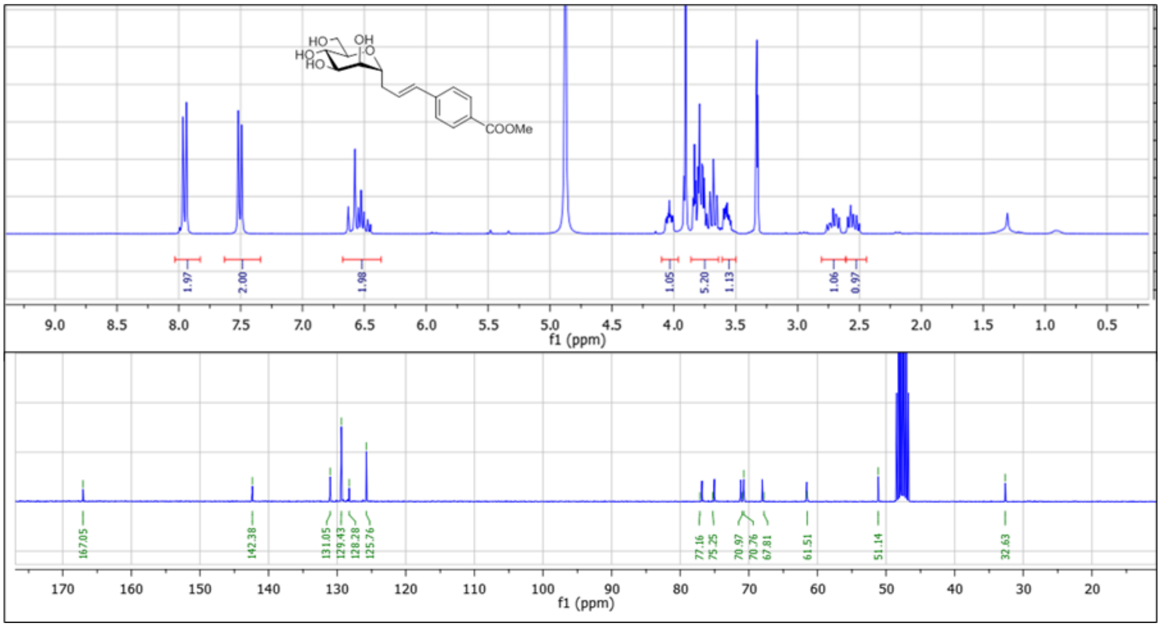

Figure S30. ^{1}H NMR and ^{13}CNMR spectrum of compound 16(CD3OD, 300 and 75MHz respectively

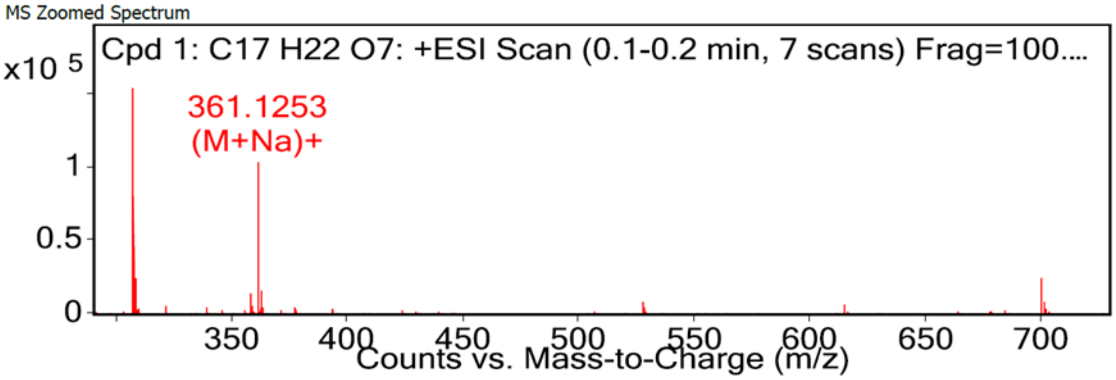

Figure S31. ESI+-HRMS spectrum of compound 16

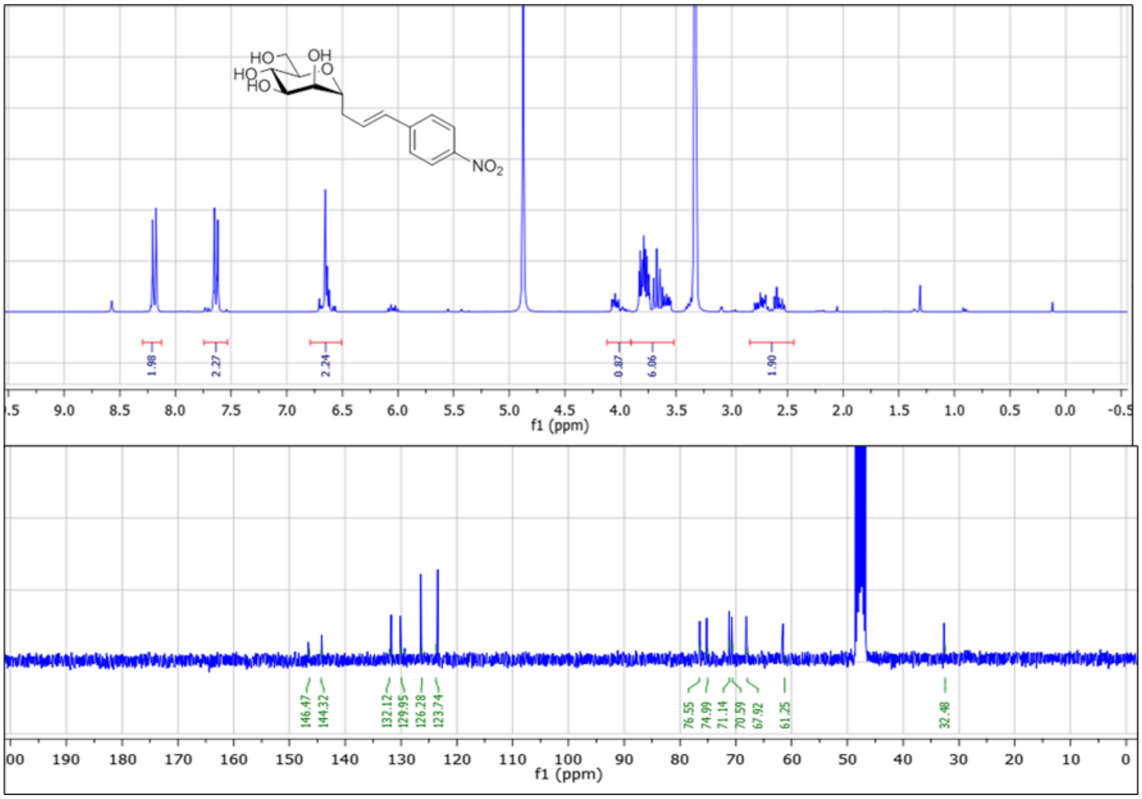

Figure S32. ^{1}H NMR and ^{13}CNMR spectrum of compound 17 (CD_3OD, 300 and 75MHz respectively

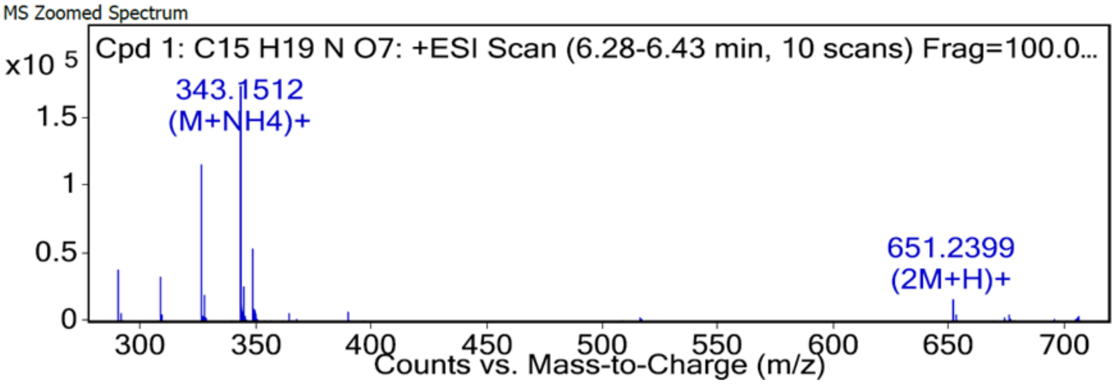

Figure S33. ESI+-HRMS spectrum of compound 17

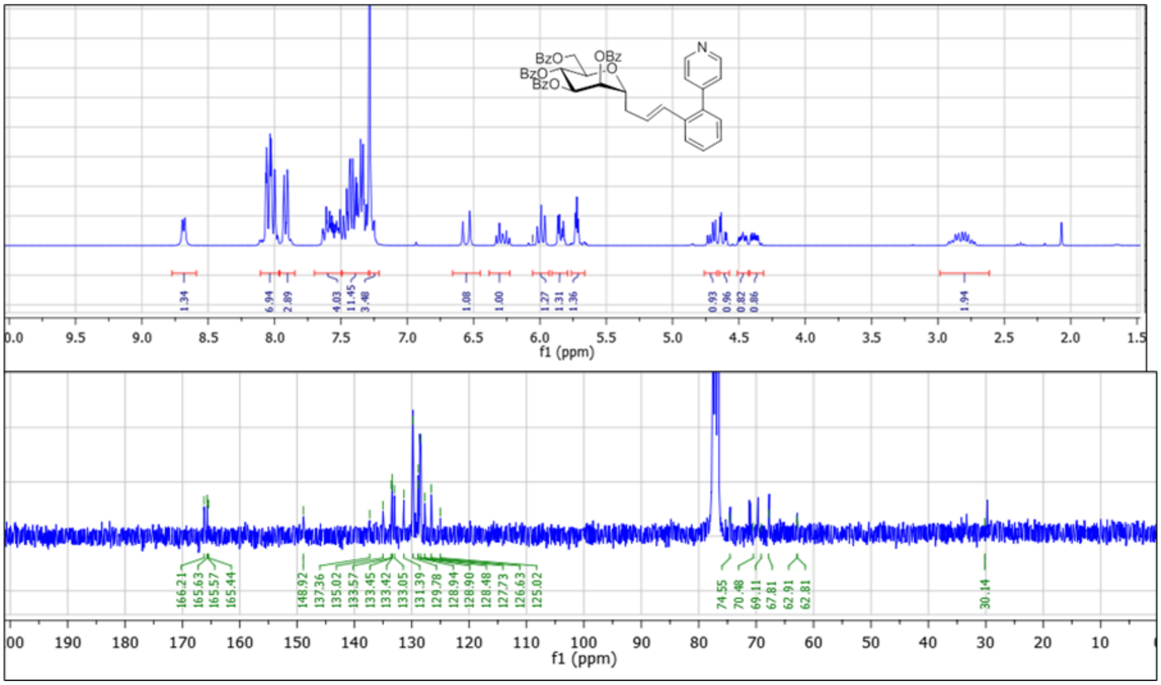

Figure S34. ¹H NMR and ¹³CNMR spectrum of compound 19 (CD_3OD, 300 and 75MHz respectively

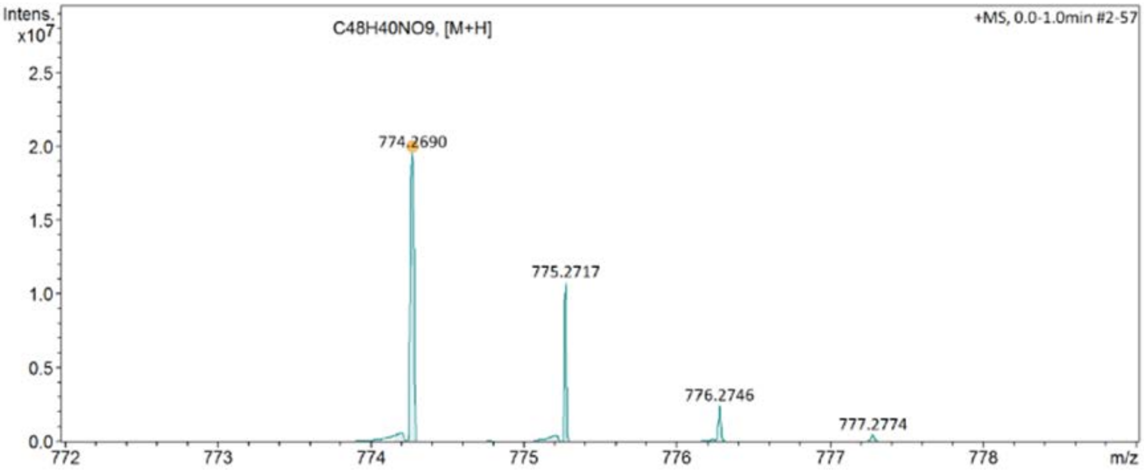

Figure S35. ESI+-HRMS spectrum of compound 19

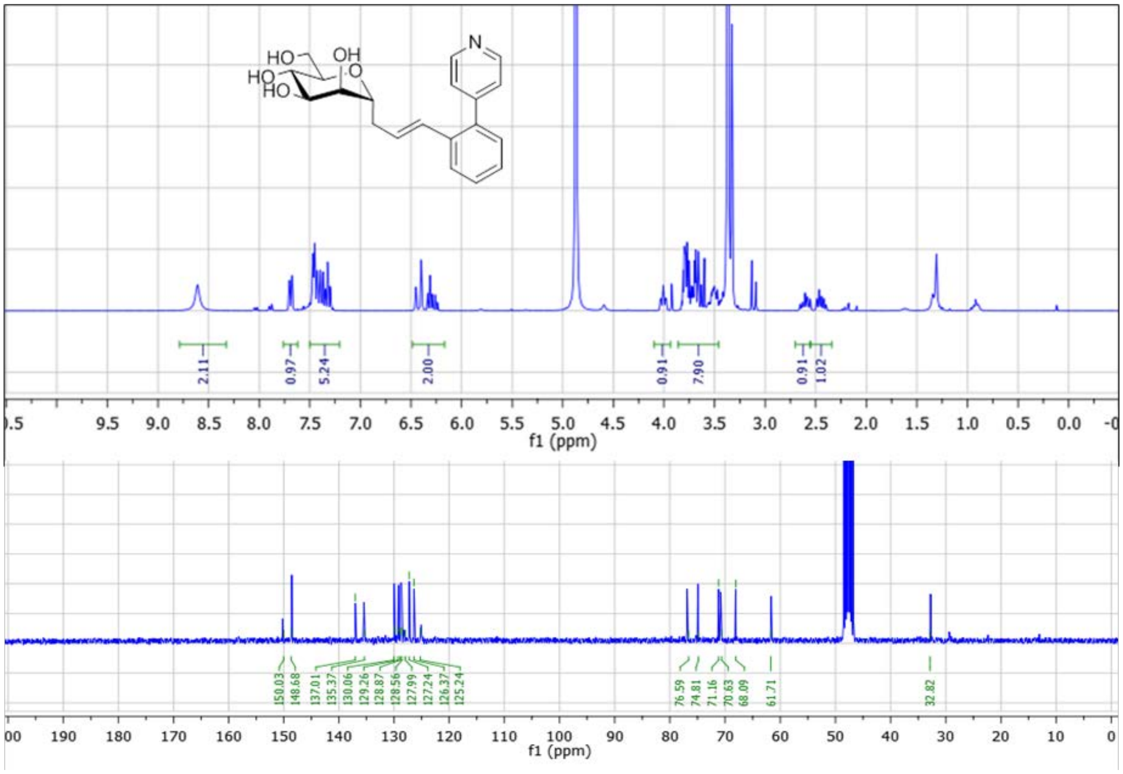

Figure S36. ^{1}H NMR and ^{13}CNMR spectrum of compound 20 (CD$_3$OD, 300 and 75MHz respectively

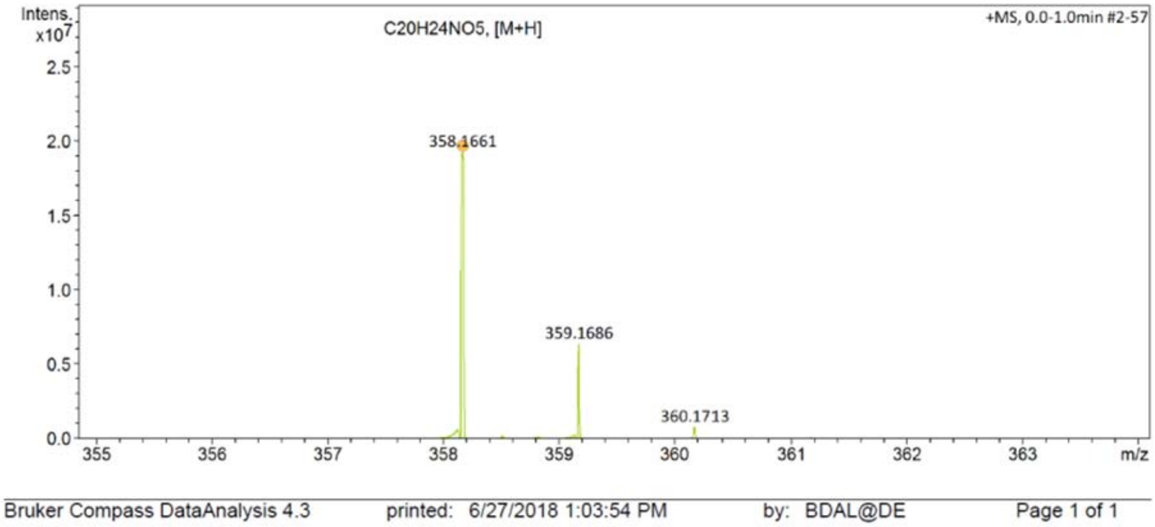

Figure S37. ESI+-HRMS spectrum of compound 20

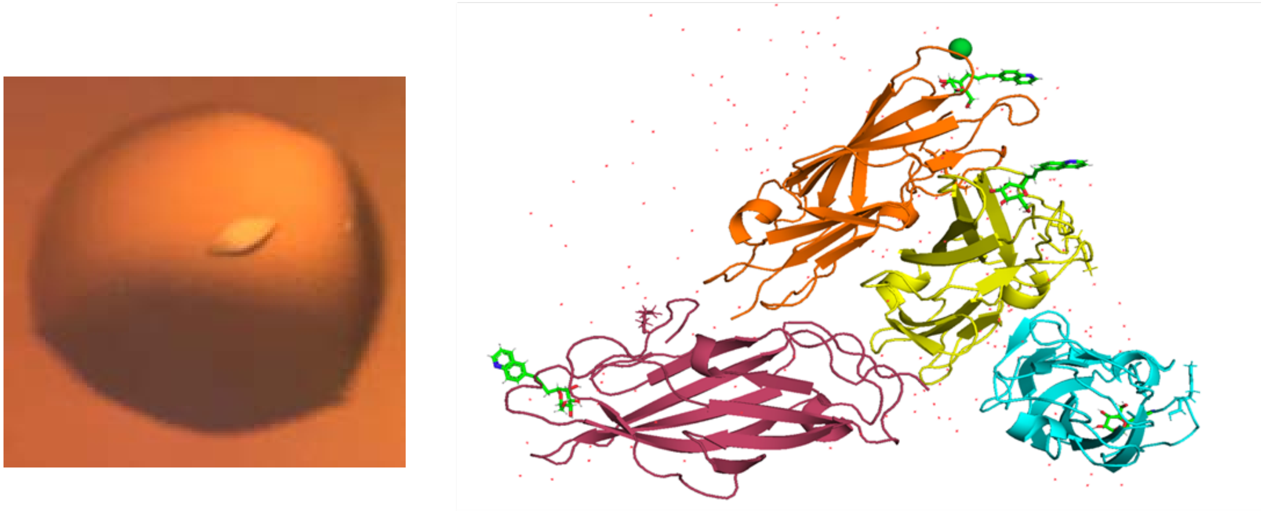

Figure S38. Single crystal of FimH bound with ligand 11

Table S1. X-ray data collection and refinement statistics.

	**FimH – (cpd 11)**
**PDB entry code**	8BVD
**Resolution range***	49.73 – 3.00 (3.18 - 3.00)
**Space group**	P 61 2 2
**Unit cell constants**	a, b = 151.784 Å, c = 225.034 Å<br>α, β = 90.00°, γ = 120.00°
**Total number of reflections**	1224300 (196951)
**Number of unique reflections**	31057 (4900)
**Multiplicity**	39.4 (40.2)
**Completeness (%)**	98.6 (98.3)
**< I/sigma(I) >**	12.41 (1.37)
**Wilson B-factor (Å2)**	46.9
**R-meas.**	0.464 (3.422)
**CC1/2**	0.996 (0.615)
**Resolution range* for refinement**	(131.45 – 3.00)
**Number of oeflections used in refinement**	27492 (34)
**Reflections used for R-free**	1439 (768)
**R-work**	0.2526 (0.301)
**R-free**	0.2906 (0.344)
**Coordinate error (Å) (maximum-likelihood based)**	0.328
**Protein residues**	632
**RMS (bonds)**	0.011
**RMS (angles)**	1.668
**Ramachandran favoured (%)**	95.03
**Ramachandran allowed (%)**	5.81
**Ramachandran outliers (%)**	0.16
**Rotamer outliers (%)**	0.20
**Clash score**	11.84
**Average B-factor (Å2)**	53.00
**macromolecules**	51.0
**ligands**	36.5

* Statistics for the highest-resolution shell are shown between parentheses.

	MeaDMan (23)				PNPMan (22)				HM (21)			
	**1**	**2**	**3**	**4**	**5**	**6**	**7**	**8**	**9**	**10**	**11**	**12**
A	200000		781.25		20000		78.13		2000		7.81	
B	100000		390.63		10000		39.06		1000		3.91	
C	50000		195.31		5000		19.53		500		1.95	
D	25000		97.66		2500		9.77		250		0.98	
E	12500		48.83		1250		4.88		125		0.49	
F	6250		24.41		625		2.44		62.5		0.24	
G	3125		12.21		312.5		1.22		31.25		PBS + FimH traceur	
H	1562.5		6.10		156.25		0.61		15.63		PBS seul	

all values nM

	Inhibitor 11				Inhibitor 20				Inhibitor 18			
	**1**	**2**	**3**	**4**	**5**	**6**	**7**	**8**	**9**	**10**	**11**	**12**
A	614,2		2,4		581,63		2.27		3481.6		13.6	
B	307,2		1,2		290.82		1,14		1740.8		6.8	
C	153,6		0,6		145.41		0.57		870.4		3.4	
D	76,8		0,3		72.70		0.28		435.2		1.7	
E	38,4		0,15		36.35		0.14		217.6		0.85	
F	19,2		0,075		18.18		0.07		108.8		0,43	
G	9,6		0,0375		9.09		0.035		54.4		PBS + FimH traceur	
H	4,8		0,0188		4.54		0,018		27.2		PBS seul	

Figure S39. A perform serial dilutiono f fimH with ligands
